# Large Rab GTPase Rab44 regulates microtubule-dependent retrograde melanosome transport in melanocytes

**DOI:** 10.1016/j.jbc.2022.102508

**Published:** 2022-09-17

**Authors:** Yuto Maruta, Mitsunori Fukuda

**Affiliations:** Laboratory of Membrane Trafficking Mechanisms, Department of Integrative Life Sciences, Graduate School of Life Sciences, Tohoku University, Sendai, Miyagi, Japan

**Keywords:** dynein, EF-hand, melanocyte, large Rab, melanosome, membrane trafficking, organelle, Rab44, retrograde transport, small GTPase, CA, constitutively active, CC, coiled-coil, cDNA, complementary DNA, EGFP, enhanced GFP, GST, glutathione-*S*-transferase, HA, hemagglutinin, LRO, lysosome-related organelle, MID, middle domain, Mreg, melanoregulin, PDD, pigment dispersion distance, RILP, Rab-interacting lysosomal protein, SR, siRNA-resistant

## Abstract

Melanosomes are melanin-containing organelles in melanocytes, and they are responsible for skin and hair pigmentation in mammals. The intracellular distribution of melanosomes is mainly determined by the balance between their anterograde transport on actin filaments and retrograde transport on microtubules. Although we have shown previously that melanoregulin and Rab36 serve as cargo receptors on melanosomes for retrograde transport, their knockdown does not completely inhibit retrograde melanosome transport, suggesting the existence of an additional cargo receptor(s) in melanocytes. In this study, we investigated the possible involvement of an atypical large Rab, Rab44, which also contains EF-hand domains and a coiled-coil domain, in retrograde melanosome transport in mouse melanocytes (Rab27A-deficient melan-ash cells). Our results showed that Rab44 localizes on mature melanosomes through lipidation of its C-terminal Rab-like GTPase domain, and that its knockdown results in suppression of retrograde melanosome transport. In addition, our biochemical analysis indicated that Rab44 interacts with the dynein–dynactin motor complex *via* its coiled-coil domain–containing middle region. Since simultaneous depletion of Rab44, melanoregulin, and Rab36 resulted in almost complete inhibition of retrograde melanosome transport, we propose that Rab44 is the third cargo receptor. We also showed that the N-terminal region of Rab44, which contains EF-hand domains, is required for both retrograde melanosome transport and its Ca^2+^-modulated activities. Our findings indicated that Rab44 is a third melanosomal cargo receptor, and that, unlike other cargo receptors previously described, its transport function is regulated by Ca^2+^.

Melanosomes are specialized melanin-containing lysosome-related organelles (so-called LROs) present in pigmented cells, including mammalian epidermal melanocytes (simply referred to as melanocytes later) (1, 2). Normal skin and hair pigmentation in mammals depends on proper melanosome transport within melanocytes and melanosome transfer from melanocytes to neighboring keratinocytes and hair matrix cells, respectively (3, 4, 5). Because the melanin contained in melanosomes is black, melanosomes can be easily identified with a conventional light microscope, and melanosomes have often been used as a model of organelle transport in the past few decades (6). Unveiling the molecular mechanism of melanosome transport is important to understanding the pathophysiology of certain hypopigmentation disorders (*i.e.*, albinism), such as Griscelli syndrome, which is caused by defects in melanosome transport (7).

The melanosomes in melanocytes are transported along two different types of cytoskeletal fibers: actin filaments and microtubules, and the intracellular distribution of melanosomes is controlled by the balance between anterograde transport (centrifugal transport toward the cell periphery) and retrograde transport (centripetal transport toward the nucleus) (6, 8). The mechanism of the actin-based melanosome transport is better understood and has been found to be mediated by a tripartite protein complex composed of the small GTPase Rab27A, its effector protein Slac2-a (also known as melanophilin), and an actin-based motor myosin Va (9, 10, 11). Functional defects in each one of these components are known to cause the hypopigmentation of Griscelli syndrome patients and the diluted coat color of murine models of Griscelli syndrome such as *ashen* (Rab27A-deficient) mice (1, 7). Melanocytes from Griscelli syndrome patients and mutant mice exhibited the same perinuclear melanosome aggregation phenotype because of a defect in melanosome transfer from microtubules to actin filaments and increased dynein-dependent retrograde transport on microtubules (simply referred to as retrograde melanosome transport later) (12, 13, 14, 15).

In contrast to the single machinery responsible for actin-based melanosome transport, both the anterograde and the retrograde melanosome transport on microtubules appear to be controlled by several distinct machineries (16, 17, 18, 19, 20), but their precise regulatory mechanisms are poorly understood. Two distinct machineries of retrograde melanosome transport have previously been reported. The first machinery consists of melanoregulin (Mreg), Rab-interacting lysosomal protein (RILP), and a dynein–dynactin subunit p150^Glued^ (19). Mreg was originally identified as a *dilute suppressor* (*dsu*) gene product whose deficiency results in the suppression of the coat color of *dilute* and *ashen* mice (12, 21) and as localizing on mature melanosomes by palmitoylation (19, 22). Mreg recruits the dynein–dynactin motor complex through RILP, and its knockdown in *ashen*-derived melanocytes (melan-ash cells) (23) causes melanosome dispersion from the perinucleus to the cell periphery (19). The second machinery consists of a protein complex that shares the RILP and p150^Glued^ of the first machinery but uses Rab36 instead of Mreg as a cargo receptor on melanosomes (20). However, since depletion of both Mreg and Rab36 in melan-ash cells does not completely inhibit retrograde melanosome transport (20), an additional as yet unidentified machinery must be present in melanocytes.

Rab44 and Rab45 (also known as Rasef) are atypical Rab family members (called large Rabs) that are characterized by containing EF-hand domains and a coiled-coil (CC) domain in the N-terminal region in addition to the C-terminal Rab-like GTPase domain (24, 25, 26) (Fig. 1*A*). It has recently been reported that Rab44 regulates the exocytosis of mast cell granules (a kind of LRO) and lysosomes (27, 28) and that Rab45 is involved in endocytic trafficking through interaction with the dynein–dynactin motor complex (29, 30). However, nothing is known about the role of large Rabs in melanosome transport in melanocytes.

**Figure 1:**
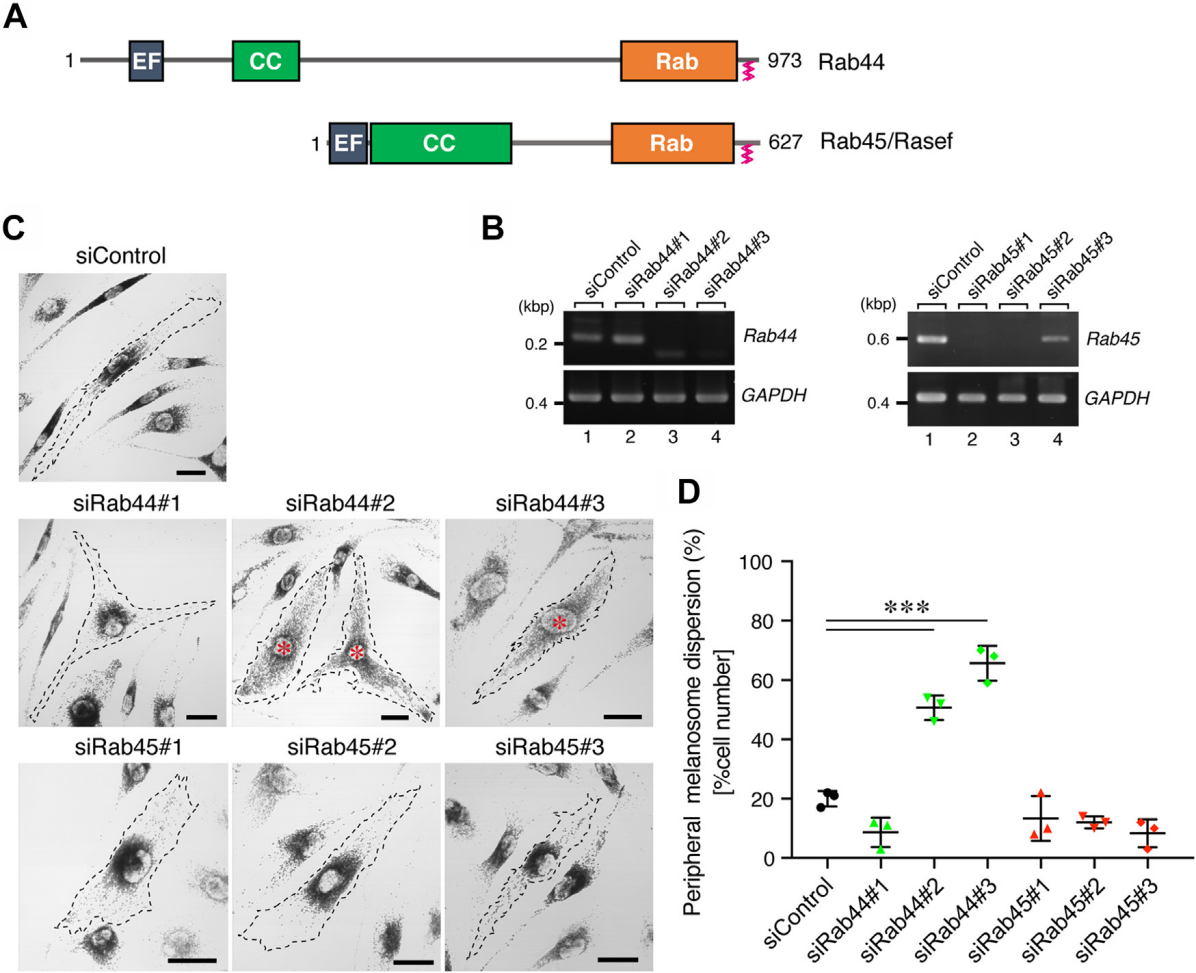
**Effect of knockdown of the large Rabs Rab44 and Rab45 on melanosome distribution in melan-ash cells.***A*, schematic representation of the mouse Rab44 and Rab45. Rab44 and Rab45 share EF-hand (EF) domains and a coiled-coil (CC) domain in addition to their Rab-like GTPase domain (Rab). The same as conventional Rabs, Rab44 and Rab45 also contain C-terminal geranylgeranylation sites. *B*, knockdown efficiency of siRNAs against *Rab44* and *Rab45* as revealed by RT–PCR analysis. GAPDH was used as a loading control (*bottom panels*). The positions of the size markers (bp, base pair) are shown on the *left*. Data shown are representative of at least three independent experiments, and similar results were obtained in each experiment. *C*, typical images of melan-ash cells (outlined with *broken lines*) that had been treated with control siRNA (siControl) *Rab44* siRNAs (siRab44#1–3) or *Rab45* siRNAs (siRab45#1–3). The *red asterisks* indicate cells showing a peripheral melanosome distribution, in contrast to the control melan-ash cells, which exhibit perinuclear melanosome aggregation. The scale bars represent 20 μm. *D*, the percentages of cells showing a peripheral melanosome distribution shown in (*C*). The error bars represent the means ± SE of the data obtained in three independent experiments (n > 25 cells in each experiment). ∗∗∗*p* < 0.001 (one-way ANOVA and Tukey’s test).

In this study, we investigated the possible involvement of large Rabs in melanosome distribution and found that knockdown of Rab44 in melan-ash cells restored the peripheral melanosome distribution but that knockdown of Rab45 did not. We also showed by coimmunoprecipitation assays that Rab44 interacts with p150^Glued^ *via* the CC domain–containing middle region and that its interaction is slightly enhanced by 10 μM Ca^2+^. Moreover, simultaneous knockdown of Rab44, Mreg, and Rab36 in melan-ash cells almost completely inhibited retrograde melanosome transport, indicating that Rab44 is a third, and presumably the last, Ca^2+^-modulated–type cargo receptor for the dynein–dynactin complex on melanosomes.

## Results

### Knockdown of Rab44, not of Rab45, in melan-ash cells restored peripheral melanosome distribution

To evaluate the possible involvement of the large Rabs Rab44 and Rab45 in retrograde melanosome transport, we turned our attention to Rab27A-deficient melan-ash cells (23), whose melanosomes are aggregated around the nucleus because of increased retrograde melanosome transport activity. Consistent with our previous studies (19, 20), knockdown of either Mreg, Rab36, or both in melan-ash cells resulted in the dispersion of melanosomes from the perinucleus to the cell periphery (Fig. S1*A*, *red asterisks*), although ∼30% of the cells still exhibited a perinuclear melanosome aggregation phenotype (Fig. S1*B*). This phenotype was most unlikely to be attributable to insufficient knockdown of Mreg, Rab36, or both, because both Mreg and Rab36 proteins in melan-ash cells mostly disappeared under our experimental conditions (Fig. S1*C*). Moreover, the observed phenotype was completely rescued by re-expression of an siRNA-resistant (SR) form of Mreg or Rab36, excluding the possibility of an off-target effect of siRNAs (Fig. S1, *D*–*F*).

To identify the remaining cargo receptor(s) on melanosomes for retrograde melanosome transport, we designed three independent siRNAs specific for Rab44 and Rab45 (named siRab44#1–3 and siRab45#1–3, respectively) and knocked down endogenous Rab44 or Rab45 in melan-ash cells. Because of unavailability of specific antibodies that worked in melan-ash cells, we were unable to detect endogenous Rab44 and Rab45 proteins under our experimental conditions (data not shown), and instead knockdown efficiency of siRNAs was evaluated by an RT–PCR analysis (Fig. 1*B*). The results showed that knockdown of Rab44 by siRab44#2 and #3 significantly increased the number of cells showing peripheral melanosome distribution (*red asterisks* in Fig. 1*C*), although ∼40% of the transfected cells still exhibited the perinuclear melanosome aggregation phenotype (Fig. 1*D*). By contrast, neither siRab44#1 nor siRab45#1–3 had any effect on the perinuclear melanosome distribution in melan-ash cells (Fig. 1, *C* and *D*). The absence of any effect of siRab44#1 on perinuclear melanosome aggregation was attributable to the insufficient knockdown of *Rab44* mRNA as revealed by an RT–PCR analysis (Fig. 1*B*, lane 2). We also investigated the effect of Rab44 knockdown (*i.e.*, knockdown with siRab44#3) on the melanosome distribution of normal melanocytes (*i.e.*, black mouse–derived melan-a cells) (31), but, the same as Mreg knockdown in melan-a cells in our previous study (19), it had no effect on peripheral melanosome distribution (Fig. S1*G*).

**Figure 2:**
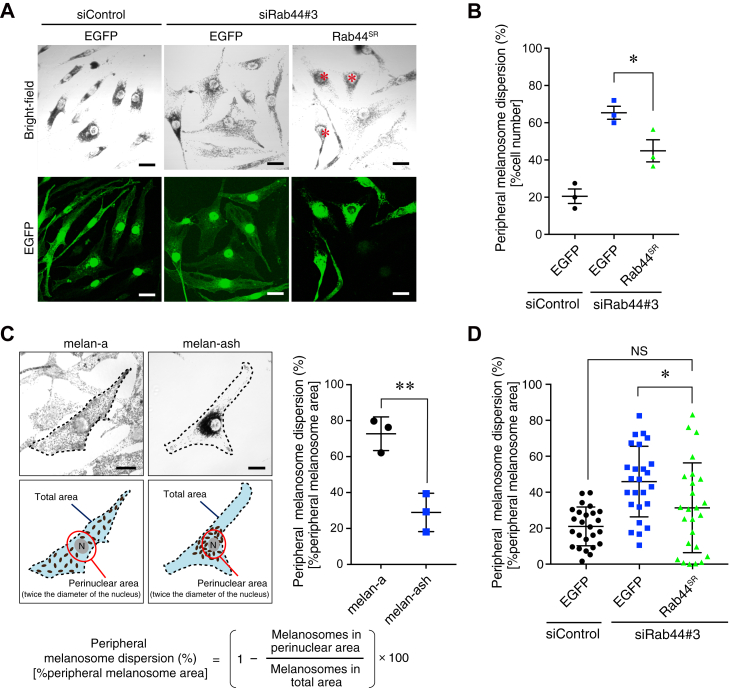
**Re-expression of EGFP-Rab44 in Rab44-depleted melan-ash cells restored perinuclear melanosome distribution.***A*, typical images of melan-ash cells stably expressing EGFP alone or siRNA-resistant EGFP-Rab44 (Rab44^SR^) that had been treated with control siRNA (siControl) or *Rab44* siRNA#3 (siRab44#3). The *red asterisks* indicate cells that exhibit perinuclear melanosome aggregation, the same as the control melan-ash cells. The scale bars represent 20 μm. *B*, the percentages of cells showing peripheral melanosome distribution in (*A*). The error bars represent the means ± SE of data obtained in three independent experiments (n > 25 cells in each experiment). *C*, definition of peripheral melanosome area as a percentage in melanocytes. Total area and perinuclear area are defined as the area within the plasma membrane (*broken lines*) and the area contained within a *circle* having twice the diameter of the nucleus (*red circles*), respectively. Melanosome signals (pixels) in the total area and perinuclear area were measured with ImageJ, and “% peripheral melanosome area” was calculated using the formula at the *bottom*. The graph on the *right* shows examples of the percent peripheral melanosome dispersion of melan-a and melan-ash cells. The error bars represent the means ± SE (n = 3 cells). ∗*p* < 0.05 (Student’s unpaired *t* test). The scale bars represent 20 μm. *D*, the percentage of peripheral melanosome dispersion/cell is shown in (*A*). “Peripheral melanosome area/cell” is defined as in (*C*) (see also the Experimental procedures section). The error bars represent the means ± SD (n > 20 cells). ∗*p* < 0.05; NS, not significant (one-way ANOVA and Tukey’s test). EGFP, enhanced GFP.

To rule out the possibility that the observed effect of Rab44 knockdown was simply attributable to an off-target effect of siRNAs, we performed a rescue experiment using an SR Rab44 mutant (Rab44^SR^) (Fig. S2*A*). Re-expression of enhanced GFP (EGFP)-tagged Rab44^SR^ in siRab44#3-treated melan-ash cells was followed by suppression of the peripheral dispersion of melanosomes in Rab44-depleted cells (Fig. 2*A*), and the percentage of cells exhibiting peripheral melanosome dispersion was significantly decreased in cells expressing EGFP-Rab44^SR^ in comparison with the control cells expressing EGFP alone (Fig. 2*B*). To analyze the knockdown phenotype in greater detail, we used peripheral melanosome dispersion calculated by the formula shown in Figure 2*C* to quantify the peripheral melanosome dispersion of single cells more accurately. Application of this formula to the melanosome distribution in melan-a cells (with predominantly peripheral melanosome distribution) and melan-ash cells (with predominantly perinuclear melanosome distribution) (Fig. 2*C*, *left images*) revealed a significant difference between the two groups (Fig. 2*C*, *right graph*). The formula was used to calculate the peripheral melanosome dispersion of each cell shown in Figure 2*A*, and the values obtained in the three groups were subjected to a statistical analysis. As shown in Figure 2*D*, the greater peripheral melanosome dispersion of the Rab44-depleted cells was significantly reduced after re-expression of EGFP-Rab44^SR^ to a level similar to the level in the control siRNA-treated cells. Taken together, these results pointed to Rab44 as a likely candidate for an additional cargo receptor for retrograde melanosome transport.

### Rab44 localizes to melanosomes *via* its RAB domain

**Figure 3:**
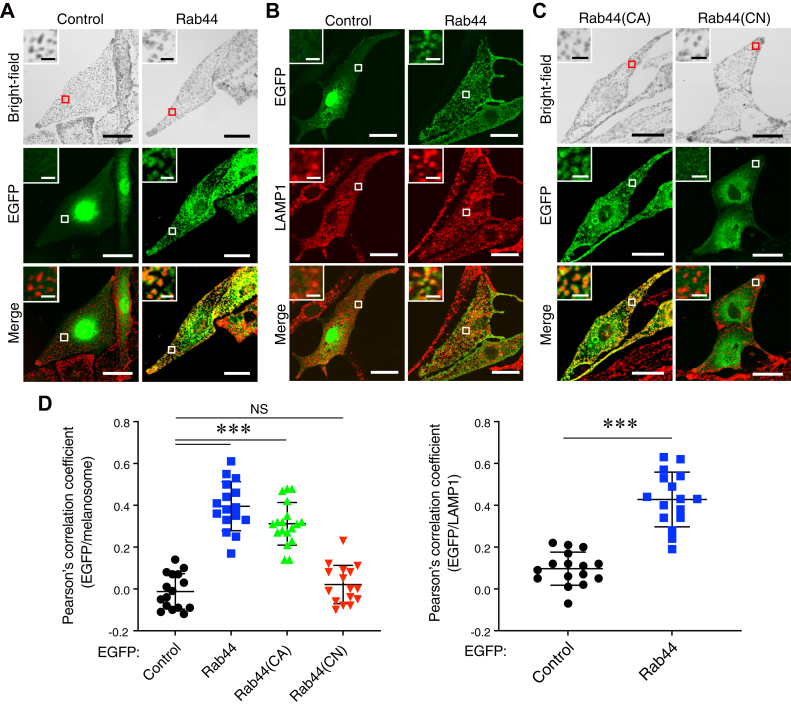
**Melanosomal and lysosomal localization of Rab44 in melanocytes.***A* and *B*, typical images of melan-a cells transiently expressing EGFP alone (control) or EGFP-tagged wildtype Rab44 (*green*). The cells in (*B*) were stained for LAMP1 (*red*). *C*, typical images of melan-a cells transiently expressing EGFP-tagged Rab44 (CA) or Rab44 (CN) (*green*) in melan-a cells. Melanosomes in the merged image panels in (*A*) and (*C*) have been pseudocolored in *red*. The *insets* show magnified views of the *boxed areas*. The scale bars represent 20 μm. *Insets*, 2 μm. *D*, quantification of the ratios of colocalization between EGFP-tagged proteins and melanosomes (*A* and *C*) or LAMP1 (*B*). Pearson’s correlation coefficients were determined (n > 15 cells), and the statistical analysis was performed by one-way ANOVA and Tukey’s test (*left graph*) or Student’s unpaired *t* test (*right graph*). ∗∗∗*p* <0.001. CA, constitutively active; CN, constitutively negative; EGFP, enhanced GFP; LAMP1, lysosomal-associated membrane protein 1; NS, not significant.

If Rab44 functions as a cargo receptor for retrograde melanosome transport, it should be localized on mature melanosomes. To identify the subcellular localization of Rab44 in melanocytes, we transiently expressed EGFP-Rab44 in melan-a cells. As expected, Rab44 colocalized with the mature melanosomes in the cell periphery (Fig. 3*A*, *inset in the bottom right panel*). Some of the EGFP-Rab44 was also colocalized with LAMP1 (lysosomal-associated membrane protein 1)-positive lysosomes (Fig. 3*B*, *inset in the bottom right panel*) as reported previously (28). Since Rab44 is a GTPase, we then investigated whether the Rab44 localized on melanosomes in an active state or an inactive state by preparing constitutively active (CA; Q844L) and constitutively negative (T799N) mutants and transiently expressing them in melan-a cells. The results showed that only the CA mutant colocalized with melanosomes, the same as the WT protein (Fig. 3*C*, *insets in the bottom panels*), although neither mutant had any effect on melanosome distribution (Fig. 3*C*, *top panels*). Thus, Rab44 is likely to localize on mature melanosomes in a GTP-dependent manner.

**Figure 4:**
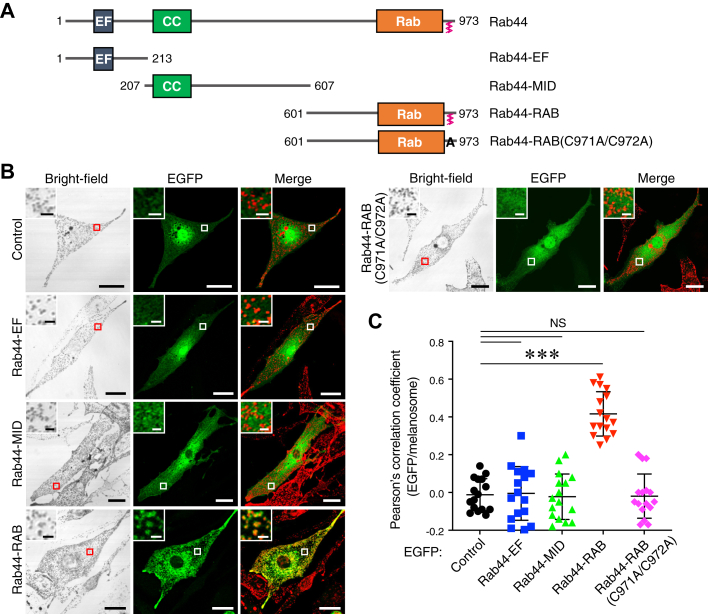
**Subcellular localization of Rab44 truncated mutants in melanocytes.***A*, schematic representation of the mouse Rab44 truncated mutants (Rab44-EF, Rab44-MID, and Rab44-RAB) and lipidation-deficient mutant (RAB(C971A/C972A)) used in this study. *B*, typical images of melan-a cells transiently expressing EGFP alone (control) or EGFP-tagged Rab44 truncated and point mutants (*green*). Melanosomes in the merged image panels have been pseudocolored in *red*. The *insets* show magnified views of the *boxed areas*. The scale bars represent 20 μm. *Insets*, 2 μm. *C*, quantification of the ratios of colocalization between EGFP-tagged proteins and melanosomes shown in (*B*). Pearson’s correlation coefficients were determined (n > 15 cells), and the statistical analysis was performed by one-way ANOVA and Tukey’s test. ∗∗∗*p* <0.001. EGFP, enhanced GFP; NS, not significant.

To identify the region responsible for the melanosomal localization of Rab44, we created three truncated mutants of Rab44, Rab44-EF, which contains an N-terminal EF-hand domain, Rab44-MID (middle domain), which contains a CC domain, and Rab44-RAB, which contains a C-terminal Rab-like GTPase domain (Fig. 4*A*). We transiently expressed these mutants with EGFP tag in melan-a cells and investigated their localizations. The results showed that only the Rab44-RAB mutant colocalized with melanosomes (Fig. 4*B*, *insets in the bottom row of the left panels*), whereas the other mutants appeared to be present in the cytoplasm. In general, conventional Rab proteins localize on organelle membranes *via* their C-terminal lipidation (*i.e.*, geranylgeranylation) (32), and Rab44 itself has been reported to localize on lysosomes *via* C-terminal lipidation (33). Thus, Rab44 should also localize on melanosomes by the same mechanism, and an additional Rab44 point mutant (named Rab44-RAB(C971A/C972A)), which lacks C-terminal lipidation sites, actually failed to exhibit melanosomal localization (Fig. 4*B*, *insets in the right panels*).

### Rab44 interacts with p150^Glued^*via* its middle region containing the CC domain

**Figure 5:**
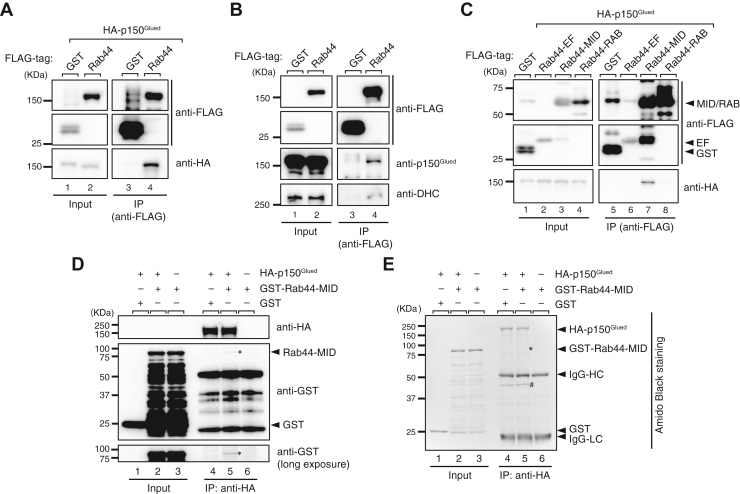
**Rab44 is associated with dynein–dynactin components.***A*, interaction between FLAG-tagged Rab44 and HA-tagged p150^Glued^ in COS-7 cells. The lower band shift of HA-p150^Glued^ was often observed in the presence of FLAG-Rab44 (lane 2, *bottom panel*). Since the molecular mass of FLAG-Rab44 and HA-150^Glued^ is almost the same, we speculate that FLAG-Rab44 artificially shifts the HA-150^Glued^ band to the lower position. *B*, interaction between FLAG-Rab44 and endogenous p150^Glued^ and the dynein heavy chain (DHC) in B16-F1 cells. *C*, interaction between HA-p150^Glued^ and the FLAG-Rab44 truncated mutants (Rab44-EF, Rab44-MID, and Rab44-RAB). Protein interactions in (*A*–*C*) were analyzed by coimmunoprecipitation (co-IP) assays using anti-FLAG tag antibody–conjugated agarose beads. *D* and *E*, direct interaction between purified GST-Rab44-MID and HA-p150^Glued^. Protein interactions were analyzed by incubating HA-p150^Glued^-bound beads with purified GST-Rab44-MID or GST alone and detection with anti-GST antibody (*D*) or Amido Black staining (*E*). The *asterisk* indicate GST-Rab44-MID bound to the beads. The *pound sign* indicates Arp1, a known binding partner of HA-p150^Glued^, which was identified by mass spectrometry analysis. The positions of the molecular mass markers (in kilodalton) are shown on the *left*. Data in (*A*–*E*) are representative of at least three independent experiments, and similar results were obtained in each experiment. GST, glutathione-*S*-transferase; HA, hemagglutinin.

Since knockdown of Rab44 in melan-ash cells restored peripheral melanosome distribution (Figs. 1*D* and 2*D*), we hypothesized that Rab44 is involved in retrograde melanosome transport on microtubules. To test our hypothesis, we investigated the interaction between Rab44 and p150^Glued^ (also known as dynactin 1), a component of the dynein–dynactin complex, which generally regulates microtubule-dependent retrograde transport, by performing coimmunoprecipitation assays in COS-7 cells. The results showed that FLAG-tagged Rab44, but not an unrelated control protein (glutathione-*S*-transferase [GST]), interacted with hemagglutinin (HA)-tagged p150^Glued^ (Fig. 5*A*). Furthermore, Rab44 expressed in B16-F1 melanoma cells was found to interact with both endogenous p150^Glued^ and the endogenous dynein heavy chain (Fig. 5*B*). We then attempted to determine the mechanism by which Rab44 interacts with the dynein–dynactin complex by performing coimmunoprecipitation assays in COS-7 cells with the three Rab44 truncated mutants described previously (Fig. 4*A*). The results showed that Rab44-MID, not Rab44-EF or Rab44-RAB, interacted with p150^Glued^ (Fig. 5*C*). The interaction between Rab44 and p150^Glued^ appeared to be direct because HA-tagged p150^Glued^ purified with HA affinity beads was able to bind to purified GST-Rab44-MID, not to GST alone (*asterisks* in Fig. 5, *D* and *E*), although under our experimental conditions, p150^Glued^ was copurified with Arp1, a known dynactin component (34) (# in Fig. 5*E*). Taken together, these results suggested that Rab44 interacts with the dynein–dynactin complex through its CC domain–containing middle region.

### Simultaneous knockdown of Rab44, Mreg, and Rab36 almost completely inhibited retrograde melanosome transport

**Figure 6:**
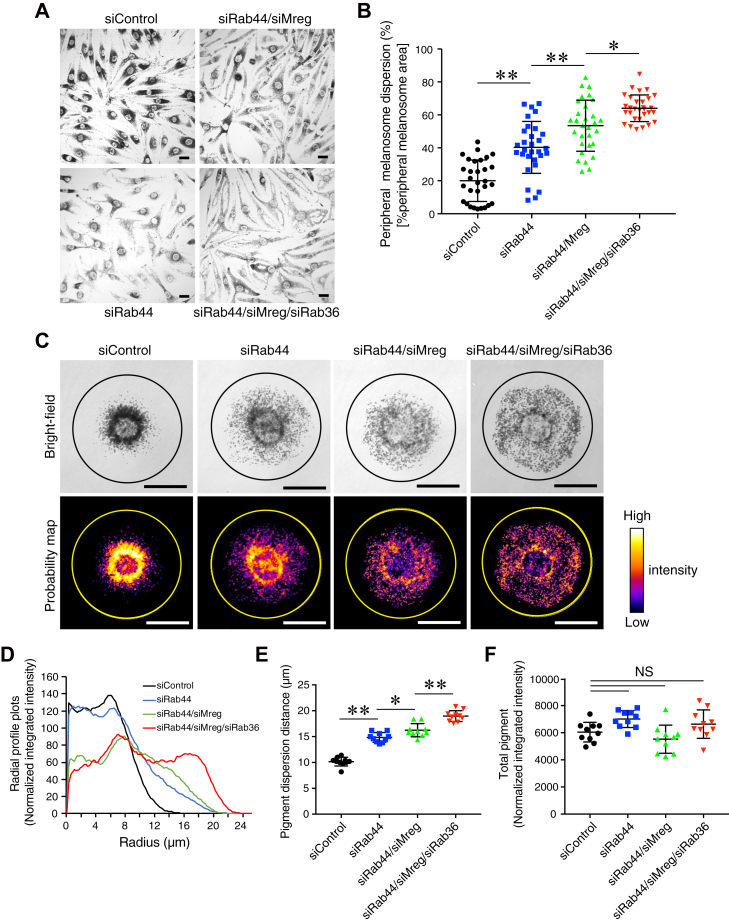
**Effect of simultaneous knockdown of Rab44, Mreg, and Rab36 on melanosome distribution in melan-ash cells.***A*, typical images of melan-ash cells that had been treated with the siRNAs indicated, that is, control siRNA (siControl), *Rab44* siRNA + *Mreg* siRNA (siRab44/siMreg), and *Rab44* siRNA + *Mreg* siRNA + *Rab36* siRNA (siRab44/siMreg/siRab36). The scale bars represent 20 μm. *B*, the percentage of peripheral melanosome dispersion/cell is shown in (*A*). The percentage of peripheral melanosome area was calculated as shown in Figure 2*C*. *C*, typical images of melan-ash cells grown on micropattern (disc-shape)-printed glass coverslips. The cells were treated with the siRNAs indicated as shown in (*A*). The melanosomes in the probability map panels have been pseudocolored with ImageJ by using look-up tables (LUTs). The diameter of the *circles* in the images is 50 μm and large enough to include the micropattern. Melanosome signal (pixel) intensity is shown on the *right*. The scale bars represent 20 μm. *D*, the radial profile plots represent the normalized integrated value of the signal intensity of subcellular melanosomes along the radius of the *circles* shown in (*C*). *E*, pigment dispersion distance (PDD) is the distance from the center of the circle containing 95% of the total signal intensity of the subcellular melanosomes in the cells shown in (*C*). *F*, total pigment means the total signal intensity of intracellular melanosomes within the *circles* in (*C*). The error bars in (*B*), (*E*), and (*F*) represent the means ± SD (n = 10 cells). ∗*p* < 0.05; ∗∗*p* < 0.01; NS, not significant (one-way ANOVA and Tukey’s test [*E*] or Dunnett’s test [*F*]).

To determine whether Rab44 functions as a remaining cargo receptor for retrograde melanosome transport in the absence of both Mreg and Rab36, we knocked down all three molecules in melan-ash cells by using specific siRNAs (Fig. S2, *B* and *C*). As expected, triple knockdown of Rab44, Mreg, and Rab36 resulted in more melanosome dispersion to the cell periphery than double knockdown of Rab44 and Mreg or knockdown of Rab44 alone did (Fig. 6, *A* and *B*). We especially noted the fact that the peripheral melanosome dispersion in the triple knockdown melan-ash cells (64.0 ± 8.1%) was comparable to that of the melan-a cells, which exhibit normal peripheral melanosome distribution (Fig. 2*C*, *right graph*, 72.7 ± 9.4%), indicating that Rab44, Mreg, and Rab36 are sufficient to mediate retrograde melanosome transport in melanocytes.

Since melanocyte morphology is not uniform, thereby precluding direct comparisons between melanosome distribution in cells of different shapes, we adopted the cell normalization technology previously established by using micropatterns (disc shape)-printed glass coverslips (35) to quantitatively investigate the melanosome distribution of Rab44-depleted cells in greater detail. When control melan-ash cells were seeded on disc-shaped–printed glass coverslips, all of them assumed a circular shape along the micropattern without any change in their perinuclear melanosome aggregation phenotype (Fig. 6*C*, *far left columns*). Intriguingly, single knockdown (Rab44 alone), double knockdown (Rab44 and Mreg), and triple knockdown (Rab44, Mreg, and Rab36) resulted in stepwise increases in melanosome dispersion to the cell periphery (Fig. 6*C*, *right three columns*). The radial profile plot of melanosomes in the knockdown cells and control cells showed that much greater melanosomes disperse in the triple knockdown cells than in the control cells (Fig. 6*D*, compare the *red and black lines*). Moreover, the pigment dispersion distance (PDD) of each cell shown in Figure 6*C* (defined as the distance from the center of the circle containing 95% of the total signal intensity of intracellular melanosomes) confirmed stepwise and significant increases in melanosome dispersion to the cell periphery (Fig. 6*E*). By contrast, the amount of melanin (or the number of melanosomes) itself was unaltered even in the knockdown cells in comparison with the control cells (Fig. 6*F*). Thus, Rab44 is likely to be the third regulator of retrograde melanosome transport in addition to Mreg and Rab36.

### Modulation of the function of Rab44 by Ca^2+^ in retrograde melanosome transport

**Figure 7:**
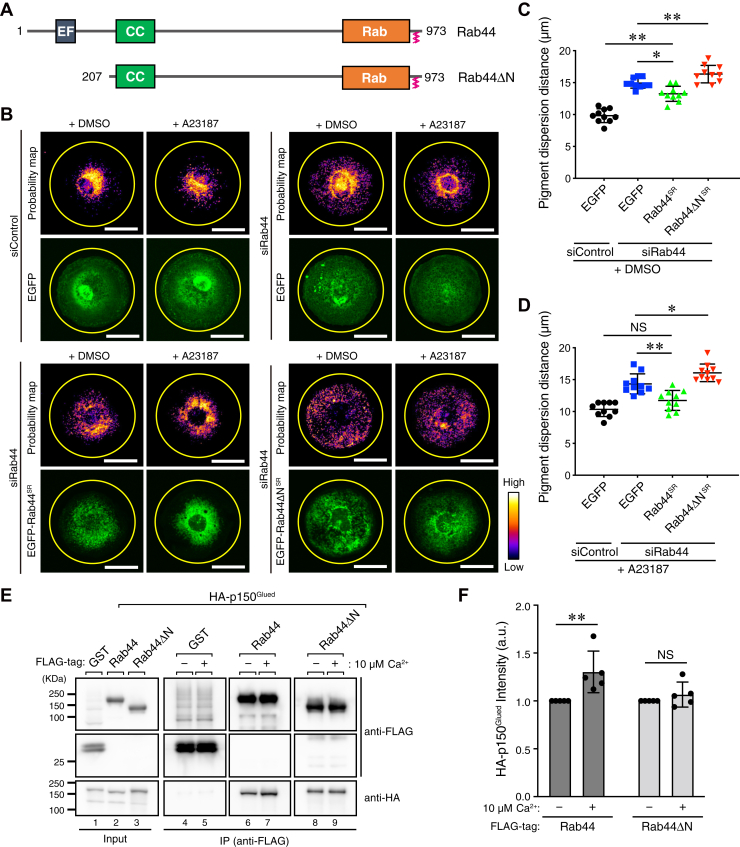
**The N-terminal region of Rab44 is required for retrograde melanosome transport in melan-ash cells.***A*, schematic representation of the mouse Rab44 and Rab44ΔN used in this study. *B*, typical images of melan-ash cells stably expressing EGFP alone, EGFP-Rab44^SR^, or EGFP-Rab44ΔN^SR^ (*green*) that had been treated with control siRNA (siControl) or *Rab44* siRNA#3 (siRab44#3). The cells grown on micropatterns (disc-shape)-printed glass coverslips were treated for 1 h with DMSO or 10 μM A23187 (Ca^2+^ ionophore). Melanosomes in the probability map panels have been pseudo-colored with ImageJ by using look-up tables (LUTs). The diameter of the circles in the images is 50 μm and large enough to include a micropattern. Melanosome signal (pixel) intensity is shown on the right. The scale bars represent 20 μm. *C* and *D*, PDD is the distance from the center of the circle containing 95% of the total signal intensity of the subcellular melanosomes in the cells shown in (*B*) that had been treated with DMSO (*C*) and A23187 (*D*). The error bars represent ±SD (n = 10 cells). ∗*p* < 0.05; ∗∗*p* < 0.01; NS, not significant (one-way ANOVA and Tukey’s test). *E*, interaction between FLAG-Rab44 or FLAG-Rab44ΔN and HA-p150^Glued^ in COS-7 cell lysates in the presence of 2 mM EGTA or 10 μM free Ca^2+^. Protein interactions were analyzed by coimmunoprecipitation (co-IP) assays with anti-FLAG tag antibody–conjugated agarose beads, followed by immunoblotting with the antibodies indicated. The positions of the molecular mass markers (in kilodalton) are shown on the *left*. Data shown are representative of five independent experiments, and similar results were obtained in each experiment. *F*, relative intensity of the HA-p150^Glued^ band shown in (*E*) in the presence or the absence of 10 μM Ca^2+^. The error bars represent the means ± SD of the data obtained in five independent experiments. ∗∗*p* < 0.01; NS, not significant (two-way ANOVA and Bonferroni test). DMSO, dimethyl sulfoxide; EGFP, enhanced GFP; HA, hemagglutinin.

Finally, we attempted to determine the function in retrograde melanosome transport of the N-terminal region of Rab44, which is not required for either p150^Glued^ binding (Fig. 5*C*) or the melanosomal localization of Rab44 (Fig. 4*B*, *left panels*). Since the N-terminal region of Rab44 contains EF-hand domains, which are well-known Ca^2+^-binding motifs, we created a Rab44 mutant lacking the N-terminal region (named Rab44ΔN in Fig. 7*A*) and investigated whether the mutant supports retrograde melanosome transport in melan-ash cells in the presence or the absence of A23187, a Ca^2+^ ionophore. After culturing Rab44-depleted melan-ash cells re-expressing EGFP alone, EGFP-Rab44^SR^, or EGFP-Rab44ΔN^SR^ on disc-shaped–printed glass coverslips (Figs. 7*B* and S3*A*), we compared their melanosome distribution and quantitatively analyzed it by PDD. In the control melan-ash cells (siControl/EGFP), the melanosomes were still aggregated in the perinuclear region even after A23187 treatment (Fig. 7*B*, *top left four panels*; and Fig. S3, *B* and *C*, *black circles*). As demonstrated above, Rab44 depletion resulted in significant melanosome dispersion to the cell periphery in comparison with the control cells (Fig. 7*B*, *top right four panels*; and Fig. 7, *C* and *D*, compare the *blue and black symbols*), and A23187 had no effect on the melanosome distribution of Rab44-depleted cells (Fig. S3*B*, *blue squares*). Re-expression of EGFP-Rab44^SR^ in the Rab44-depleted cells rescued the peripheral dispersion phenotype (Fig. 7*B*, *bottom left four panels*; Fig. 7*C*), and, intriguingly, A23187 treatment more efficiently rescued the phenotype to the level in the control cells (Fig. 7*D*, compare the *green* and *black symbols*; and Fig. S3*B*, *green triangles*). In contrast to EGFP-Rab44^SR^, EGFP-Rab44ΔN^SR^ failed to restore the perinuclear melanosome distribution of the Rab44-depleted cells irrespective of the presence of A23187 (Fig. 7*B*, *bottom right four panels*; Fig. S3*B*, *red inverted triangles*). Unexpectedly, however, EGFP-Rab44ΔN^SR^ caused more efficient melanosome dispersion to the cell periphery than Rab44 depletion did (Fig. 7, *C* and *D*, compare the *red and blue symbols*), although the EGFP-Rab44ΔN^SR^-mediated melanosome dispersion occurred independently of A23187 (Fig. S3*B*, *red inverted triangles*). These results suggested that EGFP-Rab44ΔN^SR^ can cause melanosome dispersion to the cell periphery independently of Rab44 depletion. As expected, many EGFP-Rab44ΔN^SR^-expressing melan-ash cells exhibited peripheral melanosome distribution (Fig. S3, *D* and *E*).

Based on the aforementioned results, we hypothesized that the interaction between Rab44 and p150^Glued^ is enhanced by Ca^2+^, which promotes retrograde melanosome transport under A23187-stimulated conditions more efficiently than under resting conditions (Fig. 7, *B* and *D*). To test our hypothesis, we performed coimmunoprecipitation assays in the presence of 2 mM EGTA (*i.e.*, Ca^2+^-free conditions) or 10 μM Ca^2+^, which almost correspond to increased Ca^2+^ concentrations under physiological conditions. The results showed that the interaction between Rab44 and p150^Glued^ was weakly but significantly enhanced by Ca^2+^, whereas, consistent with the fact that Rab44ΔN lacks EF-hand domains, no enhancement of the interaction between Rab44ΔN and p150^Glued^ was observed (Fig. 7, *E* and *F*).

## Discussion

In the present study, we identified Rab44 as a third cargo receptor on melanosomes that regulates microtubule-dependent retrograde melanosome transport. In contrast to the cargo receptors previously reported, that is, Mreg and Rab36 (19, 20), Rab44 does not require a linker protein such as RILP to recruit the dynein–dynactin complex and instead interacts with p150^Glued^ *via* its own middle region containing the CC domain (Fig. 5*C*). However, Rab44 localizes to melanosomes by a lipidation mechanism (Fig. 4) similar to the mechanism of Rab36 and Mreg localization (19, 20, 22). Although Rab44 is recruited to melanosomes in a GTP-dependent manner, overexpression of the active CA form of Rab44 in normal melanocytes (melan-a cells) did not affect peripheral melanosome distribution (Fig. 3*C*), unlike overexpression of the CA form of Rab36, which induces perinuclear melanosome aggregation (20). This finding suggests that the interaction between Rab44 and the dynein–dynactin complex itself is insufficient to support retrograde melanosome transport and that an additional factor may enhance or trigger Rab44-mediated retrograde melanosome transport.

We speculate that Ca^2+^ may be that factor, because the Rab44–p150^Glued^ interaction was significantly enhanced by a physiological concentration of Ca^2+^ (Fig. 7, *E* and *F*), and re-expression of Rab44 in Rab44-depleted melan-ash cells rescued the peripheral dispersion phenotype in the presence of A23187 more efficiently than in the control resting conditions (Fig. 7, *B*–*D*). Consistent with our speculation, deletion of the N-terminal region containing the EF-hand domains impaired the Ca^2+^ sensitivity of the Rab44 functions (Fig. 7, *B* and *F*), even though Rab44ΔN still contains both the p150^Glued^-binding region and melanosome-targeting region. These findings taken together suggest that the N-terminal region of Rab44 is essential for the regulation or modulation of retrograde melanosome transport. Thus, it is not surprising that Rab44ΔN acts as a dominant negative construct by trapping the dynein–dynactin complex, which consequently inhibits retrograde melanosome transport and promotes peripheral melanosome distribution in melan-ash cells (Fig. S3, *D* and *E*). While this article was being prepared for publication, there was a report that Rab44 regulates kinesin-1-dependent secretory granule translocation in mast cells, although it remains to be determined whether Rab44 itself interacts with kinesin-1 (36). However, our preliminary data, at least the data obtained in melanocytes by coimmunoprecipitation assays (unpublished data), did not show interaction between Rab44 and Kif5b, the major isoform of kinesin-1 heavy chain in melanocytes (17). Since inhibition of retrograde transport indirectly promotes anterograde transport on microtubules, in the future, it will be interesting to investigate whether Rab44 regulates retrograde transport of LROs in other LRO-containing cells, including mast cells.

The physiological significance of the presence of three cargo receptors, Mreg, Rab36, and Rab44, for retrograde melanosome transport in melanocytes is an open question that needs to be addressed in future studies. In contrast to Mreg and Rab36, Rab44 contains a unique N-terminal region, which contains Ca^2+^-sensitive EF-hand domains (Fig. 1*A*), but the involvement of Ca^2+^ in melanosome distribution or even the significance of retrograde melanosome transport itself in mammalian melanocytes is hardly understood. By contrast, in fish and amphibian melanophores (pigment cells that produce melanin), both anterograde and retrograde melanosome transport play an essential role in the regulation of body color change by switching between melanosome dispersion and aggregation, respectively (37). This switching process is finely tuned by hormones such as α-melanocyte-stimulating hormone that alter the intracellular cAMP level and activity of several protein kinases, including PKA (38, 39). Intriguingly, epinephrine stimulation has been shown to both decrease the intracellular cAMP level and increase the intracellular Ca^2+^ concentration and to lead to melanosome aggregation in certain types of melanophores (39, 40). Thus, since Rab44 is widely conserved in vertebrates, the function of Rab44 in retrograde melanosome transport may be more important in fish and amphibian melanophores than in mammalian melanocytes. Keratinocytes exposed to ultraviolet radiation secrete hormones and cytokines that modulate melanogenesis in mammalian melanocytes, and these substances may alter the intracellular Ca^2+^ concentration, which would affect melanosome distribution (*e.g.*, Fig. 7*D*) by modulating Rab44-mediated retrograde melanosome transport in melanocytes. Further extensive research will be needed to investigate this possibility.

In summary, we have investigated microtubule-dependent retrograde melanosome transport in mouse melanocytes and succeeded in identifying the atypical large GTPase Rab44 as a third cargo receptor on melanosomes. We have also shown that retrograde melanosome transport is almost entirely regulated by three independent cargo receptors, Mreg, Rab36, and Rab44, the last of which alone is regulated by Ca^2+^ *via* its EF-hand–containing N-terminal region. We thus propose that during skin pigmentation and body color changes, Rab44 regulates retrograde melanosome transport in response to extracellular stimuli such as hormones and cytokines that raise the intracellular Ca^2+^ concentration.

## Experimental procedures

### Materials

The antibodies, plasmids, siRNAs, and primers used in this study are summarized in Table S1. Unless otherwise specified, all other materials used in this study were of analytical grade or of the highest grade commercially available.

### Molecular cloning of mouse Rab44 and preparation of its truncated mutants

Mouse Rab44 complementary DNA (cDNA) was amplified from Marathon-Ready adult brain and testis cDNAs (Takara Bio) by PCR using the specific pairs of oligonucleotides shown in Table S1. cDNAs encoding CA and constitutively negative mutants (Q844L and T799N, respectively), a C971A/C972A mutant (Fig. 4*A*), an SR mutant, and truncated mutants (EF, MID, RAB, and ΔN; Figs. 4*A* and 7*A*) of Rab44 were prepared by the standard molecular biology techniques using the specific oligonucleotides shown in Table S1. These Rab44 cDNAs and mouse p150^Glued^ cDNA (19) were subcloned into appropriate vectors (Table S1). The Rab44 and p150^Glued^ expression plasmids are available from RIKEN BioResource Research Center in Japan (https://dnaconda.riken.jp/search/depositor/dep005893.html; catalog no.: RDB19675–RDB19681).

### Cell cultures, transfections, and stable expression of EGFP-Rab44 mutants

Melan-a cells and melan-ash cells, immortal mouse melanocyte cell lines derived from a black mouse and a *Rab27A*-deficient mouse (*ashen* mouse), respectively, were obtained from the Wellcome Trust Functional Genomics Cell Bank at St George’s, University of London, and they were cultured as described previously (23, 31). B16-F1 cells (obtained from the American Type Culture Collection) and COS-7 cells were cultured at 37 °C in Dulbecco’s modified Eagle’s medium (catalog no.: 044-29765; FUJIFILM Wako Pure Chemical) supplemented with 10% fetal bovine serum, 100 U/ml penicillin G, and 100 μg/ml streptomycin in a 5% CO_2_ incubator. Retrovirus production and infection were performed essentially as described previously (41, 42). Melan-ash cells stably expressing EGFP-Rab44^SR^ mutants were selected by exposure to 2 μg/ml puromycin for 2 days. Cells were transfected with plasmid DNAs and siRNAs by using Lipofectamine 2000 and RNAiMAX (Thermo Fisher Scientific), respectively, according to the manufacturers’ instructions.

### Melanosome distribution assays

Three days after transfecting siRNAs (final concentration of 10 nM) into melan-ash cells, the percentage of cells showing peripheral melanosome distribution was calculated after a manual cell count. Cells in which more than 50% of the melanosomes were present around the nucleus were classified as “aggregated” (43), and the rest of the cells were classified as “dispersed” (Figs. 1*D*, 2*B*, and Fig. S1*B* and *F*). For the more detailed analyses shown in Figures 2*D* and 6*B*, and Fig. S3*E*, the peripheral melanosome dispersion of melanocytes was calculated by dividing the number of melanosome signals (pixels) dispersed outside the perinuclear region (twice the diameter of the nucleus) by the number of signals (pixels) in the total cell area (named “%peripheral melanosome area”; see the formula in Fig. 2*C*). The number of pixels in each area was calculated by using ImageJ software (version 2.1.0/1.53c; National Institutes of Health).

### Immunofluorescence analysis

Three days after transfecting plasmids into melan-a cells, the cells were fixed with 4% paraformaldehyde for 10 min, permeabilized with 0.05% saponin for 30 min, and blocked with 1% bovine serum albumin in PBS for 30 min. The cells were then stained with anti-LAMP1 antibody (1/100 dilution; 1.5 h), followed by visualization with Alexa Fluor 594-conjugated secondary antibody. The stained cells were examined for fluorescence with a confocal fluorescence microscope (FluoView 1000-D; Evident/Olympus) through an objective lens (60× magnification, numerical aperture 1.40; Evident/Olympus) and with FluoView software (version 4.1a; Evident/Olympus). The images were processed with ImageJ software.

### Micropattern cell culture and melanosome distribution analysis

Three days after transfecting siRNAs (final 10 nM) into melan-ash cells or melan-ash cells stably expressing EGFP-Rab44^SR^ or EGFP-Rab44ΔN^SR^, the cells were transferred into 6-well plates containing CYTOOchips (DC-L-A; CYTOO) and cultured for 16 h. The cells were then treated with dimethyl sulfoxide or A23187 (final concentration of 10 μM) (catalog no.: 019-0111; FUJIFILM Wako Pure Chemical) for 1 h and fixed with 4% paraformaldehyde for 10 min. The fixed cells were examined with a confocal fluorescence microscope as described previously, and the images captured were processed with ImageJ software. The radial profiles shown in Figure 6*D* were obtained by inverting each bright-field image and pseudocoloring the melanosomes by using Fire look-up tables. Their signal (pixel) intensities were normalized and integrated by using the radial profile plugin for ImageJ, and the values obtained were plotted along a circle (radius = 25 μm) that includes a micropattern (*circles* in Figs. 6*C* and 7*B*). PDD in Figures 6*E* and 7, *C* and *D*, and Fig. S3*B* was calculated as the distance from the center of the circle containing 95% of the total signal intensity of the intracellular melanosomes. Total pigment in Figure 6*F* and Fig. S3*C* was calculated as the sum of the signal intensities of the melanosomes in the circles.

### RT–PCR analysis

Total RNA was isolated from siRNA-treated melan-ash cells with TRI Reagent (Molecular Research Center), and reverse transcription was performed by using ReverTra Ace -α- (Toyobo) according to the manufacturer’s instructions. The cDNAs of *Rab45* and *GAPDH* were amplified by PCR performed with KOD plus DNA polymerase (Toyobo), and the cDNA of *Rab44* was amplified by a nested PCR performed with KOD plus Neo DNA polymerase (Toyobo). The authenticity of the PCR products was verified by DNA sequencing them. The specific primers used for the amplification of the *Rab44*, *Rab45*, and *GAPDH* cDNAs are summarized in Table S1.

### Coimmunoprecipitation assays and direct binding assays

For the coimmunoprecipitation assays in COS-7 cells, cells that had been cotransfected for 2 days with pEF-FLAG-Rab44 (or FLAG-Rab44 truncated mutants) and pEF-HA-p150^Glued^ were lysed with a lysis buffer (50 mM Hepes–KOH, pH 7.2, 150 mM NaCl, 1 mM MgCl_2_, 5% glycerol, and 0.5% NP-40 supplemented with cOmplete, EDTA-free protease inhibitor cocktail [Roche]). The cell lysates were incubated for 16 h at 4 °C with anti-FLAG tag antibody–conjugated agarose beads (Sigma–Aldrich). After washing the beads three times with a washing buffer (50 mM Hepes–KOH, pH 7.2, 150 mM NaCl, 1 mM MgCl_2_, and 0.1% NP-40), proteins bound to the beads were analyzed by 10% SDS-PAGE followed by immunoblotting with the appropriate horseradish peroxidase–conjugated antibodies summarized in Table S1. Immunoreactive bands were visualized by enhanced chemiluminescence, and images were captured by a ChemiDoc Touch Imaging System (Bio-Rad). For the Ca^2+^-dependent interaction between Rab44 and p150^Glued^ in Figure 7*E*, a 10 μM free calcium solution was prepared by using the Ca^2+^ chelator EGTA. The ratio of Ca^2+^ and EGTA used to prepare the solution was calculated using the Ca–EGTA calculator (44). Based on the results of the calculations, 0.1 M CaCl_2_ (Nacalai Tesque) and 0.1 M EGTA·2Na (Nacalai Tesque) were added to the cell lysates, and they were incubated for 16 h at 4 °C with anti-FLAG tag antibody–conjugated agarose beads. Proteins bound to the beads were analyzed as described previously.

For the coimmunoprecipitation assays in B16-F1 cells, cells that had been transfected for 2 days with pEF-FLAG-Rab44 were lysed with the lysis buffer. The cell lysates were incubated for 16 h at 4 °C with anti-FLAG tag antibody–conjugated agarose beads. After washing the beads three times with the washing buffer, proteins bound to the beads were analyzed by 10% SDS-PAGE followed by immunoblotting with the appropriate antibodies (Fig. 5*B* and Table S1).

For the direct binding assays, GST-Rab44-MID was affinity purified with glutathione Sepharose 4B (Cytiva) as described previously (45), and HA-p150^Glued^ transiently expressed in COS-7 cells was affinity purified with anti-HA tag–conjugated agarose beads (Sigma–Aldrich). The beads coupled with HA-p150^Glued^ were incubated for 2 h at 4 °C with purified GST-Rab44-MID or GST alone as a control in PBS. After washing the beads with the washing buffer three times, the GST-Rab44-MID bound to the beads was analyzed by 10% SDS-PAGE followed by immunoblotting with anti-GST antibody and then Amido Black staining (Fig. 5, *D* and *E*).

### Statistical analysis

The statistical analysis was performed by one-way ANOVA followed by Tukey’s test or Dunnett’s test (for multiple comparison) and two-way ANOVA followed by Bonferroni test or Student’s unpaired *t* test (for comparison between two samples), using GraphPad Prism 9 software (GraphPad Software, Inc). All quantitative data are expressed as the means ± SD or SE. The *asterisks* in the graphs indicate *p* values (∗*p* < 0.05; ∗∗*p* < 0.01; and ∗∗∗*p* < 0.001). NS, not significant (*p* > 0.05).

## Data availability

The data generated are included in the main text file and supporting information.

## Supporting information

This article contains supporting information (46, 47, 48, 49, 50).

## Conflict of interest

The authors declare that they have no conflicts of interest with the contents of this article.
