# Supplementary material for: Large Rab GTPase Rab44 regulates microtubule-dependent retrograde melanosome transport in melanocytes
Source: J Biol Chem. 2022 Sep 17;298(11):102508. doi: 10.1016/j.jbc.2022.102508 (PMC9586991; doi:10.1016/j.jbc.2022.102508)
Supplement: Supplemental Figures S1–S3 and Table S1 [file mmc1.pdf]

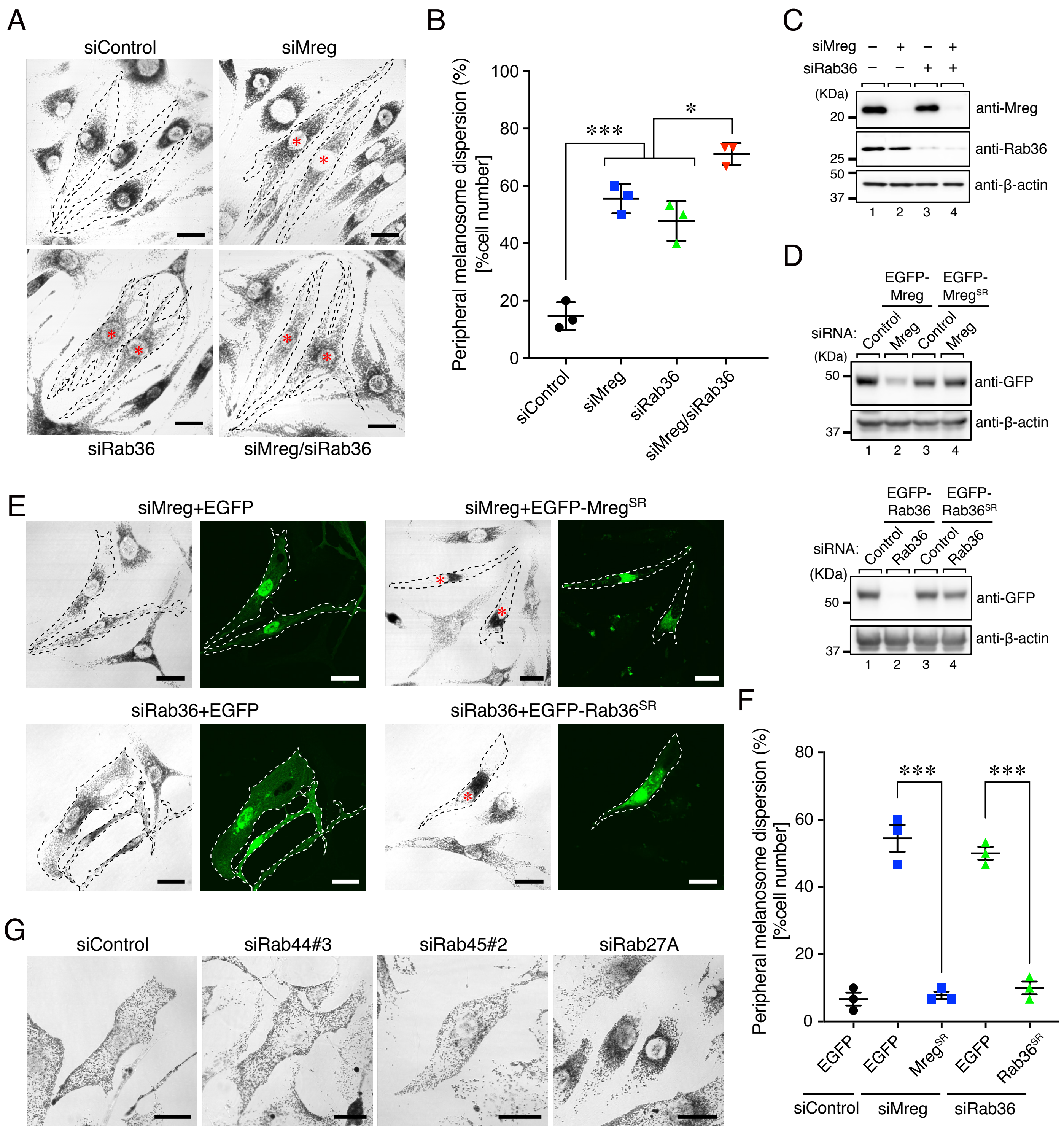
A
siControl
siMreg
siRab36
siMreg/siRab36
B
Peripheral melanosome dispersion (%) [%cell number]
100
80
60
40
20
0
***
*
siControl
siMreg
siRab36
siMreg/siRab36
C
siMreg – + – +
siRab36 – – + +
(KDa)
20
25
50
37
anti-Mreg
anti-Rab36
anti-β-actin
1 2 3 4
D
EGFP-Mreg
EGFP-MregSR
siRNA: Control Mreg Control Mreg
(KDa)
50
37
anti-GFP
anti-β-actin
1 2 3 4
EGFP-Rab36
EGFP-Rab36SR
siRNA: Control Rab36 Control Rab36
(KDa)
50
37
anti-GFP
anti-β-actin
1 2 3 4
E
siMreg+EGFP
siMreg+EGFP-MregSR
siRab36+EGFP
siRab36+EGFP-Rab36SR
F
Peripheral melanosome dispersion (%) [%cell number]
80
60
40
20
0
***
***
EGFP
EGFP
MregSR
EGFP
Rab36SR
siControl
siMreg
siRab36
G
siControl
siRab44#3
siRab45#2
siRab27A

Figure S1. Effect of double knockdown of Mreg and Rab36 on melanosome distribution in melan-ash cells and effect of knockdown of large Rabs on melanosome distribution in melan-a cells. (A) Typical images of melan-ash cells (outlined with broken lines) that had been treated with the siRNAs indicated, i.e., control siRNA (siControl), *Mreg* siRNA (siMreg), *Rab36* siRNA (siRab36), and *Mreg* siRNA + *Rab36* siRNA (siMreg/siRab36). The red asterisks indicate the cells showing peripheral melanosome distribution, in contrast to the control melan-ash cells, which exhibit perinuclear melanosome aggregation. Scale bars, 20 µm. (B) The percentage of cells showing peripheral melanosome distribution shown in (A). The error bars represent the means ± S.E. of the data obtained in three independent experiments (n > 25 cells in each experiment). *, $p < 0.05$; ***, $p < 0.001$ (one-way ANOVA and Tukey's test). (C) Knockdown efficiency of Mreg and Rab36 proteins as revealed by immunoblotting with the antibodies indicated. (D) An siRNA-resistant (SR) mutants of Mreg and Rab36 are shown. siRNAs and pGEFP-C1 plasmids were co-transfected into COS-7 cells, and their lysates were analyzed by immunoblotting with the antibodies indicated. Data in (C) and (D) are representative of at least two independent experiments, and similar results were obtained in each experiment. (E) Typical images of $Mreg^{SR}$- or $Rab36^{SR}$-expressing melan-ash cells (outlined with broken lines; *, rescued cells) that had been treated with the siRNAs indicated. (F) The percentage of cells showing peripheral melanosome distribution shown in (E). The error bars represent the means ± S.E. of the data obtained in three independent experiments (n > 25 cells in each experiment). ***, $p < 0.001$ (one-way ANOVA and Tukey's test). (G) Typical images of melan-a cells that had been treated with control siRNA (siControl), *Rab44* siRNA (siRab44#3), *Rab45* siRNA (siRab45#2), or *Rab27A* siRNA (siRab27A). Scale bars, 20 µm.

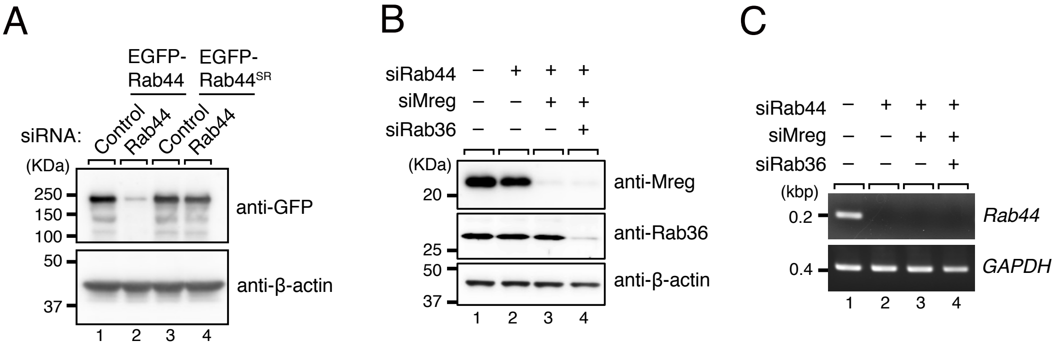

Figure S2. Knockdown efficiency of siRNAs against Rab44, Mreg, and Rab36 in melan-ash cells. (A) Knockdown efficiency of *Rab44* siRNA (siRab44#3) toward recombinant mouse Rab44 and an siRNA-resistant mutant of Rab44 ($Rab44^{SR}$) in COS-7 cells (see Figs. 1C and 2A). (B) Knockdown efficiency of *Mreg* siRNA (siMreg) and *Rab36* siRNA (siRab36) as revealed by immunoblotting with specific antibodies (see Fig. 6A). (C) Knockdown efficiency of *Rab44* siRNA (siRab44#3) as revealed by RT-PCR analysis (see Fig. 6A). Data in (A)–(C) are representative of at least two independent experiments, and similar results were obtained in each experiment.

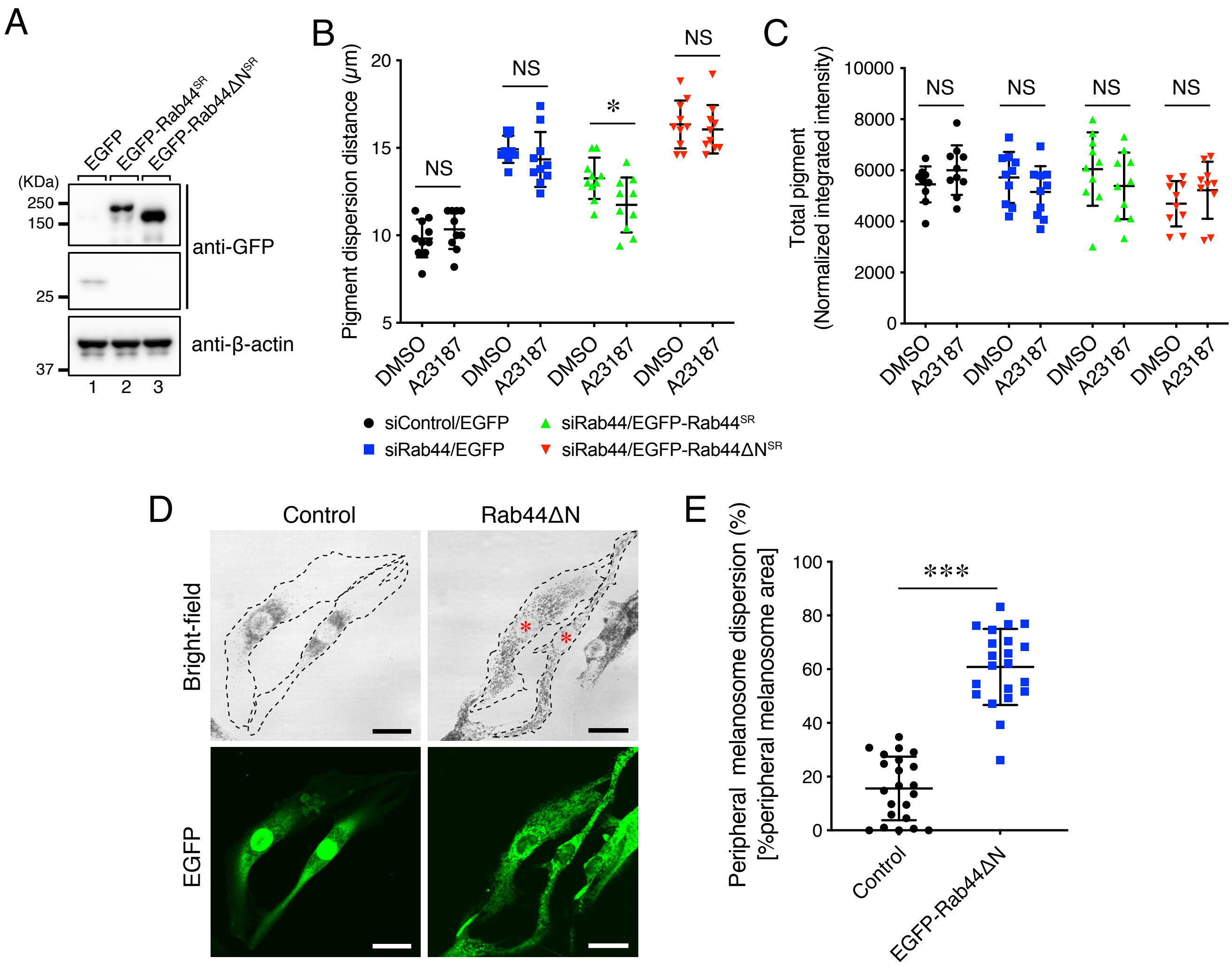

Figure S3. Re-expression of EGFP-Rab44ΔN in Rab44-depleted melan-ash cells failed to restore perinuclear melanosome distribution. (A) The protein expression levels of EGFP alone, EGFP-Rab44^{SR}, and EGFP-Rab44ΔN^{SR} in the melan-ash cells shown in Fig. 7B. The cell lysates were analyzed by immunoblotting with the antibodies indicated. The positions of the molecular mass markers (KDa) are shown on the left. The blots shown are representative of two independent experiments. (B) The PDD is the distance from the center of the circle containing 95% of the total signal intensity of the subcellular melanosomes in the cells shown in Fig. 7B. (C) Total pigment means the total signal intensity of the intracellular melanosomes within the circles in Fig. 7B. The error bars in (B) and (C) represent the means ± S.D. (n = 10 cells). *, $p < 0.05$; NS, not significant (two-way ANOVA and Bonferroni test). (D) Typical images of melan-ash cells stably expressing EGFP alone or EGFP-Rab44ΔN (Rab44ΔN^{SR}). The red asterisks indicate the cells (outlined with broken lines) showing hyper-dispersion of melanosomes. Scale bars, 20 μm. (E) The percentage of peripheral melanosome dispersion/cell is shown in (D). The percentage of peripheral melanosome area was calculated as shown in Fig. 2C. The error bars represent the means ± S.D. (n > 20 cells). ***, $p < 0.001$ (Student's unpaired *t*-test).

Table S1. List of materials used in this study

Oligonucleotides	Sequence (5'-to-3' direction)	Source
siRNAs		
mouse *Rab44* siRNA#1	TCATGACGCTTTTGCTACT	Nippon Gene (Toyama, Japan)
mouse *Rab44* siRNA#2	ACCTGCTGGTAGACAACAA	Nippon Gene (Toyama, Japan)
mouse *Rab44* siRNA#3	GAGATCAGCTTGCTTTTGA	Nippon Gene (Toyama, Japan)
mouse *Rab45* siRNA#1	GGAGTTGATTTCCAAATGA	Nippon Gene (Toyama, Japan)
mouse *Rab45* siRNA#2	CGATGTTACTTGTGAGAAA	Nippon Gene (Toyama, Japan)
mouse *Rab45* siRNA#3	GCAAATCCATGATTTAACA	Nippon Gene (Toyama, Japan)
mouse *p150^{Glued}* siRNA	Not available	Thermo Fisher (Waltham, MA) MSS203510
mouse *Mreg* siRNA	TCCGTATTCCTCCTTTGGA	Nippon Gene (Toyama, Japan)
mouse *Rab36* siRNA	AGACTAGCCTCATTCACAG	(46) *EMBO Rep.* (2013) 14, 450-457
mouse *Rab27A* siRNA	AAGAGAGTGGTGTACAGAG	(46) *EMBO Rep.* (2013) 14, 450-457

Primers for mutagenesis and construction		
Rab44-Met	GGATCCATGGAGAAAGGAAAGGGAGT	Nippon Gene (Toyama, Japan)
Rab44-EF-C	GCGTCGACTCACTTTCTCAGGGTCCATGCCAG	Nippon Gene (Toyama, Japan)
Rab44-MID-N	TGCGGATCCCTGGCATGGACCCTGAGAAAG	Nippon Gene (Toyama, Japan)
Rab44-MID-C	GCGTCGACTCACTTAGCATGGCTCTCCTGCCT	Nippon Gene (Toyama, Japan)
Rab44-RAB-N	TGCGGATCCAGGCAGGAGAGCCATGCTAAG	Nippon Gene (Toyama, Japan)
Rab44-RAB-C	GCGTCGACTCAGTGGCAGCAGCCAGCTCTCTT	Nippon Gene (Toyama, Japan)
Rab44-RAB-C-Δstop	AGCGAATTCCTTAGCATGGCTCTCCTGCCT	Nippon Gene (Toyama, Japan)
Rab44-SR-5'	TTCAGGGACCAACTAGCATTCGAGGCTGAG	Nippon Gene (Toyama, Japan)
Rab44-SR-3'	CTCAGCCTCGAATGCTAGTTGGTCCCTGAA	Nippon Gene (Toyama, Japan)
Rab44-Q844L-5'	GACACAGCTGGACTCGAGAGGTACCAC	Nippon Gene (Toyama, Japan)
Rab44-Q844L-3'	GTGGTACCTCTCGAGTCCAGCTGTGTC	Nippon Gene (Toyama, Japan)
Rab44-T799N-5'	AATGTGGGCAAGAATTCATTCCTACAC	Nippon Gene (Toyama, Japan)
Rab44-T799N-3'	GTGTAGGAATGAATTCTTGCCCACATT	Nippon Gene (Toyama, Japan)
Rab44-C971A/C972A-3'	GCGTCGACTCAGTGGGCGGCGCCAGCTC	Nippon Gene (Toyama, Japan)
Mreg-SR-5'	AACAATCCATACTCATCATTCGGCGCGACT	Nippon Gene (Toyama, Japan)
Mreg-SR-3'	AGTCGCGCCGAATGATGAGTATGGATTGTT	Nippon Gene (Toyama, Japan)

RT-PCR primers		
GAPDH-forward	ACCACAGTCCATGCCATCAC	Eurofins Genomics (Tokyo, Japan)
GAPDH-reverse	TCCACCACCCTGTTGCTGTA	Eurofins Genomics (Tokyo, Japan)
Rab44-forward Nested 1st	ATGCCTGTTCAGGTGGAGAGC	Eurofins Genomics (Tokyo, Japan)
Rab44-reverse Nested 1st	TCAGTGGCAGCAGCCAGCTCT	Eurofins Genomics (Tokyo, Japan)
Rab44-forward Nested 2nd	TTACTGGCTCGACTGTCTGC	Eurofins Genomics (Tokyo, Japan)
Rab44-reverse Nested 2nd	TGCATCTTAAGTGACCGAGCC	Eurofins Genomics (Tokyo, Japan)
Rab45-forward	GGTGAGGGCAGCACTCTTAG	Eurofins Genomics (Tokyo, Japan)
Rab45-reverse	TCCCGTGCAAGATGGAGAAC	Eurofins Genomics (Tokyo, Japan)
Mreg-forward	GGATCCAATTTATGGAGCATGCCT	(19) *J Cell Sci.* (2012) 125, 1508-1518
Mreg-reverse	GTCGACCATTTGGCTAGCAATCTT	(19) *J Cell Sci.* (2012) 125, 1508-1518
Rab36-Met	CGGATCCATGAGGTCCTCTTGGACCCC	(47) *J. Biol. Chem.* (2003) 278, 15373-15380
Rab36-stop	TTAACAGCAGCCTAGGCCGG	(47) *J. Biol. Chem.* (2003) 278, 15373-15380

Plasmids	RIKEN BioResource Research Center Cat#	Source
pEF-FLAG-GST		(48) *Cell Struct. Funct.* (2020) 45, 45-55
pEF-FLAG-Rab44 (long form)		This study
pEF-FLAG-Rab44ΔN		This study
pEF-HA/T7-p150^{Glued}	RBD19675	(19) *J Cell Sci.* (2012) 125, 1508-1518
pEGFP-C1		Clontech-Takara Bio (Shiga, Japan) #6084-1
pEGFP-C1-Rab44 (long form)	RBD19676 (RBD17957: short form)	This study
pEF-FLAG-Rab44^{SR} (long form)		This study
pEGFP-C1-Rab44-Q844L (CA)	RBD19677	This study

pEGFP-C1-Rab44-T799N (CN)	RBD19678	This study
pEGFP-C1-Rab44-EF	RBD19679	This study
pEGFP-C1-Rab44-MID	RBD19680	This study
pEGFP-C1-Rab44-RAB	RBD19681	This study
pEGFP-C1-Rab44-RAB-C971A/C972A		This study
pEGFP-C1-Rab36		(20) *J. Biol. Chem.* (2012) 87, 28619-28631
pEGFP-C1-Rab36^{SR}		(20) *J. Biol. Chem.* (2012) 87, 28619-28631
pEGFP-C1-Mreg	RDB15182	(19) *J Cell Sci.* (2012) 125, 1508-1518
pEGFP-C1-Mreg^{SR}		This study
pGEX-4T-3		Merck (Darmstadt, Germany) #GE28-9545-52
pMRX-IRES-puro-EGFP		(49) *J. Biol. Chem.* (2003) 278, 36005–36012
pMRX-IRES-puro-EGFP-Rab44^{SR}		This study
pMRX-IRES-puro-EGFP-Rab44ΔN^{SR}		This study

Antibodies	Dilution	Source
Anti-FLAG tag (M2)-HRP mouse mAb	IB (1/1,000 dilution)	Sigma-Aldrich (St. Louis, MA) A8592
Ant-GFP rabbit pAb	IB (1/1,000 dilution)	MBL (Nagoya, Japan) 598
anti-HA tag (3F10)-HRP rat mAb	IB (1/500 dilution)	Roche (Rotkreuz, Switzerland) 12013819001
anti-β-actin-HRP mouse mAb	IB (1/5,000 dilution)	Proteintech (Rosemont, IL) HRP-60008
anti-Dynein Heavy Chain rabbit pAb	IB (1/500 dilution)	Proteintech (Rosemont, IL) 12345-1-AP
anti-p150^{Glued} (1/p150Glued) mouse mAb	IB (1/500 dilution)	BD Biosciences (Franklin Lakes, NJ) 610474
anti-GST (B-14) mouse mAb	IB (1/10,000 dilution)	Santa Cruz Biotechnology (Dallas, TX) sc-138
anti-LAMP1 (1D4B) rat mAb	IF (1/100 dilution)	BD Biosciences (Franklin Lakes, NJ) 553792
anti-Mreg guinea pig pAb	IB (0.35 µg/ml)	This study
anti-Rab36 guinea pig serum	IB (1/1,000 dilution)	(50) *Biol. Open* (2014) 3, 803-814

IB, immunoblot; IF, immunofluorescence; mAb, monoclonal antibody; pAb, polyclonal antibody.
